# Impacts of Artificial Underground Reservoir on Groundwater Environment in the Reservoir and Downstream Area

**DOI:** 10.3390/ijerph16111921

**Published:** 2019-05-30

**Authors:** Ya Sun, Shi Guo Xu, Ping Ping Kang, Yan Zhao Fu, Tian Xiang Wang

**Affiliations:** 1 School of Hydraulic Engineering, Dalian University of Technology, Dalian 116024, China; sunya_nj@mail.dlut.edu.cn (Y.S.); yzfu@mail.dlut.edu.cn (Y.Z.F.); tianxiang@dlut.edu.cn (T.X.W.); 2 School of Water Conservancy, North China University of Water Resources and Electric Power, Zhengzhou 450045, China; ppkang@outlook.com

**Keywords:** artificial underground reservoir, environmental impact, groundwater flow, nitrogen pollutant distribution

## Abstract

Artificial underground reservoirs have changed the hydrological cycle from its natural condition. This modification may trigger a series of negative environmental effects both at the local and regional levels. This study investigated the impact of the Wanghe artificial underground reservoir on groundwater flow and quality in the reservoir and its downstream area. Wanghe is a typical artificial underground reservoir scheme in China, which assumes the dual function of fresh-water preservation and control of seawater intrusion. The groundwater flow pattern has changed after the reservoir construction, and the water level in the reservoir rose rapidly. Evaluation of long-term groundwater level fluctuation suggested that the reservoir deprived the downstream aquifer of the runoff, which it received under the natural flow regime. A preliminary isotopic evaluation using ^3^H was developed to understand the groundwater flow and renewal rates in the study area. The uniform distribution of tritium levels in the reservoir indicated that the stored water was well-mixed in both horizontal and vertical directions. The intervention on groundwater circulation also made differences in groundwater renewal rates between stored and downstream water. Field investigations on groundwater nitrogen pollution showed that the construction of the artificial underground reservoir resulted in nitrate accumulation in the stored water. Agriculturally derived nitrate was the largest contributor, and ${\mathrm{NO}}_{3}^{-}$ concentration varied considerably over time due to fertilization and irrigation activities, rainfall, and denitrification. ${\mathrm{NO}}_{3}^{-}$-N distributed homogeneously in the reservoir, which was attributed to the construction of the subsurface dam, land use pattern and artificial groundwater flow.

## 1. Introduction

China has been facing severe water shortages and pollution due to improper water resource development, utilization, and management [1,2]. In recent decades, underground storage via artificial underground reservoirs has emerged as a promising strategy for augmenting water reserves [3,4,5]. Artificial underground reservoirs (sometimes named groundwater reservoir/underground reservoirs) are a water storage, supply, and regulation system that makes use of a natural aquifer as a water storage space. An artificial underground reservoir generally consists of artificial recharge facilities, a subsurface dam, and groundwater extraction facilities [6,7]. It directs excess surface water into the aquifer through artificial recharge and arrests groundwater flow by constructing a subsurface dam (if needed) to augment groundwater reserves for subsequent use. Compared with traditional surface reservoirs, artificial underground reservoirs have the advantages of limited evaporation loss, no siltation, less susceptibility to pollution, no dam failure disaster, no land submergence, and resettlement associated with surface dams [8,9,10,11].

Artificial underground reservoirs have met with great success in China in the last few decades [12]. Over 100 artificial underground reservoirs have been built in various parts of the country to increase groundwater reserves and solve environmental problems that resulted from groundwater level decline. Most of the schemes are large-scale projects with a storage capacity of tens to hundreds of Mm^3^. Appendix A Table A1 lists some typical artificial underground reservoir projects, and there are a great number of potential sites [13,14]. However, artificial underground reservoir development is accompanied by environmental risks due to the lack of evaluation of possible impacts on groundwater environment. Previous studies have mostly concentrated on the positive roles of artificial underground reservoirs in increasing water reserves [15,16,17] and relieving seawater intrusion [18,19,20]. At present, there is a lack of in-depth explorations of the negative environmental effects caused by artificial underground reservoirs in the zone of benefit and the downstream region. Artificial underground reservoirs intervene in water circulation. Artificial recharge (infiltration or injection techniques, e.g., spreading basin, injection wells) modifies the hydrological cycle by adding to the storage and flow of groundwater. Particularly, the groundwater dam abruptly and strongly changes the natural behavior of the aquifer, which blocks the two-way exchange of groundwater [21,22]. These modifications may trigger a series of negative environmental effects in both local and downstream areas [23].

Groundwater environmental impacts are most evident in groundwater level and flow patterns. The diversion of surface water for groundwater recharge results in a reduction of downstream flow. According to the practical experiences of a small-scale subsurface dam in Africa, the subsurface dam can deprive the downstream aquifer of the benefit of ground water seepage, which it receives under the natural flow regime [24,25,26,27]. For the large-scale projects in China, the resulting downstream flow decrease may be more severe. In addition, the elevation of the reservoir water-level to the vicinity of the surface can lead to waterlogging and soil salinization due to incremental evaporation [28]. Small-scale underground dams are widely used in rural areas in northeastern Brazil, where salt build-up in the upstream area of the dam is the major problem over several usage cycles [29].

The deterioration of the water quality of artificial underground reservoirs is the least predictable and remains difficult to remedy. Groundwater recharge is the main factor that leads to contamination of the receiving aquifer. Local agricultural runoff, industrial effluents, and domestic sewage can be potential pollution sources, as there is no reservoir-related resettlement. Recharge with source waters of impaired quality will introduce microbial and chemical constituents into the receiving aquifer [30]. The biochemical and geochemical reactions between the source water, resident groundwater, and aquifer materials could result in water quality change [31,32]. In addition, artificial recharge can leach contaminating substances from the unsaturated zone to groundwater and extend the range of pollution plumes [33]. Although a fraction of the pollutants can be removed by soil self-purification or water exchange, the rest of them accumulate within the reservoir due to repeated use of stored water. The extent of the impact is associated with property of the dam, pumping of stored water, rainfall variation, character of aquifer, land use pattern, etc. [34,35]. Long-term monitoring of the nitrate concentration in the artificial underground reservoir of the Sunagawa subsurface dam before and after the dam’s construction indicated that the subsurface dam and pumping stored water greatly affected groundwater flow and water mixing in the reservoir area. The ${\mathrm{NO}}_{3}^{-}$-N concentration decreased gradually and harmonized with the decrease in cultivation acreage [36,37]. Yoshimoto, S. et al. [38] proposed a numerical model consisting of a water balance sub-model and nitrogen balance sub-model to predict the long-term changes of groundwater nitrate in the reservoir area of the Komesu subsurface dam. The results showed that, when the annual rainfall decreases at a rate of 5.5% per 100 years, the minimum groundwater level decreases, and the ${\mathrm{NO}}_{3}^{-}$-N level in the artificial underground reservoir increases. Lalehzari, R. et al. [39,40] evaluated the impact of a semi-pervious subsurface dam on the fate of groundwater nitrate in the Shahrekord aquifer. Though nitrate concentration rose slightly in the stored water and decreased in the downstream region, there was no considerable difference before and after the dam construction in short times. For China, artificial underground reservoir quality could be at a much greater risk due to the overuse of chemical fertilizers and dense population. The Longhe artificial underground reservoir, located in Lushun, China, which was constructed in 2000, fell into disuse a few short years later due to serious groundwater pollution [6].

It is necessary to give insight into the impact of artificial underground reservoirs on the groundwater environment in the affected areas. The Wanghe artificial underground reservoir is a typical artificial underground reservoir in China, which undertakes water storage and control of seawater intrusion. This study takes the Wanghe artificial underground reservoir as a case study, to reveal the impacts on groundwater environment in the reservoir and its downstream area under the influence of the Wanghe underground reservoir’s construction. We have accomplished the following work: (1) Analyzed the groundwater flow pattern change and groundwater level fluctuation before and after reservoir construction; conducted groundwater tritium concentration to reveal the groundwater movement and exchange characteristics; calculated the groundwater renewal rate with the well-mixed reservoir model. (2) Conducted field surveys on groundwater nitrogen pollutant (${\mathrm{NO}}_{3}^{-}$-N, ${\mathrm{NO}}_{2}^{-}$-N and ${\mathrm{NH}}_{4}^{+}$-N) distribution in the study area; investigated pollution sources and calculated pollution loads; analyzed the temporal-spatial distribution of groundwater N pollutants; identified the main reasons for the enrichment of nitrate pollutants in the reservoir area. The findings of this paper can be scaled up since the problems that the Wanghe artificial underground reservoir face now are likely being encountered by other artificial underground reservoirs in China.

## 2. Materials and Methods 

### 2.1. Geology and Hydrogeology

**Figure 1:**
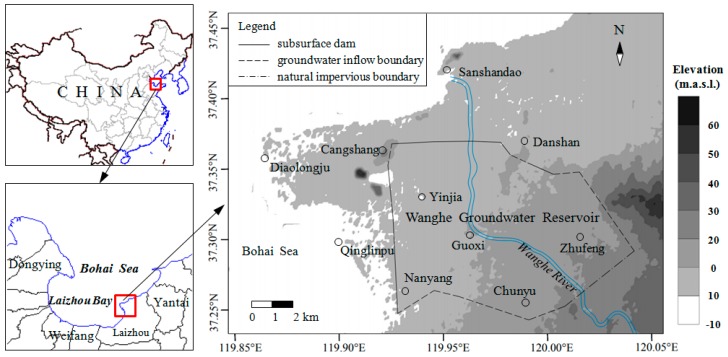
Location of study area and Wanghe artificial underground reservoir.

The research area is located in the northwest of Laizhou city (Shandong Province, China) at latitude 119.82° E–120.05° E and longitude 37.16° N–37.43° N, with an area of approximately of 200 km^2^ (Figure 1). It is a plain lying on the coast of the Bohai Sea in the west and north and bounded by hills in the east. The terrain slopes gently from southeast to northwest. It has a semi-humid monsoon climate. The temperature averages 13.1 ℃ annually. The mean annual precipitation is 579.1 mm, and over 70% of the rainfall is concentrated within June to September. Wanghe River, a seasonal river fed by monsoon flows from southeast to northwest through the area, and finally into the sea. Groundwater occurs mainly in the alluvial deposits and is recharged by precipitation and river runoff. The groundwater flow direction is identical to that of the surface relief, from southeast to northwest.

### 2.2. Land Use and Artificial Underground Reservoir Project

**Figure 2:**
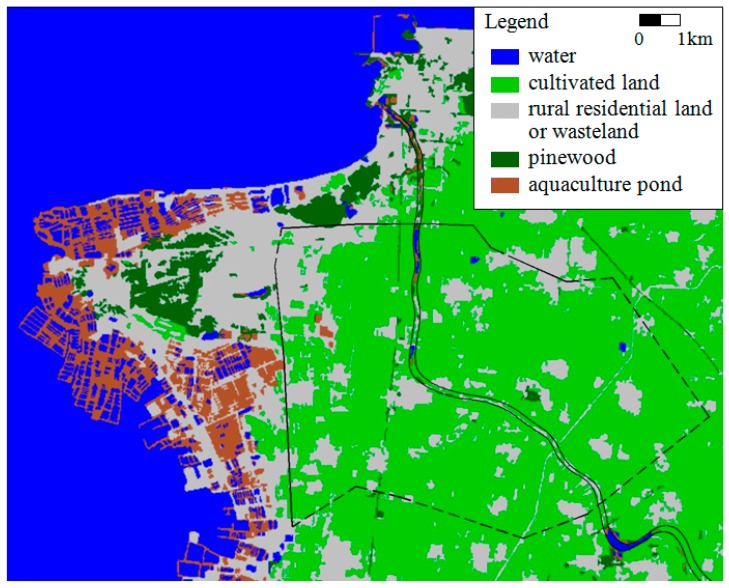
Land use pattern of the study area.

As indicated in Figure 2, land use for cultivated land is dominant within the reservoir area, which makes up over 60% of the total reservoir area, followed by village settlements. The downstream area consists mainly of cultivated land, rural residential land, pine wood land, and wasteland. The offshore area has been reclaimed for mariculture.

Since the 1970s, the extensive groundwater exploitation has resulted in a steep decline in groundwater level, which induced an aggressive landward movement of seawater. The Wanghe artificial underground reservoir was implemented to meet the high irrigation water consumption and combat saltwater intrusion in 2002. The reservoir covers an area of 68.49 km^2^, with a total storage capacity of 56.93 million m^3^ and effective storage capacity of 32.73 million m^3^. 

**Figure 3:**
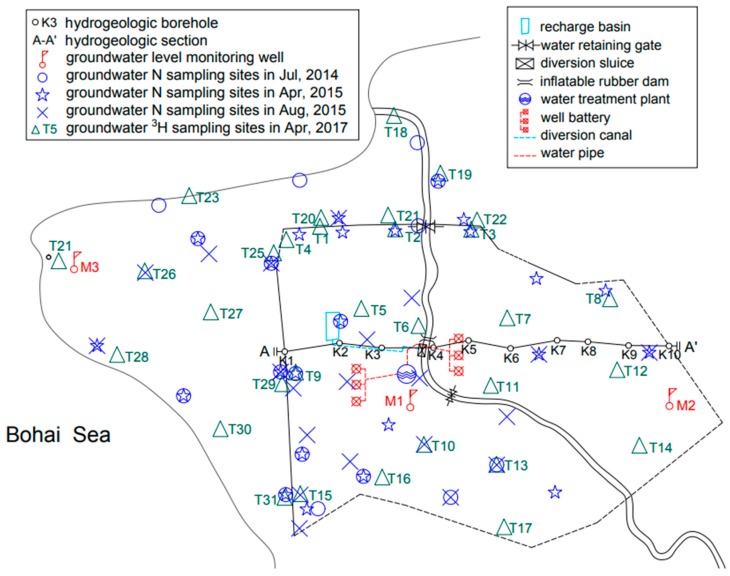
Layout of the Wanghe artificial underground reservoir, location of groundwater sampling sites, groundwater level monitoring wells, and hydrogeological section.

**Figure 4:**
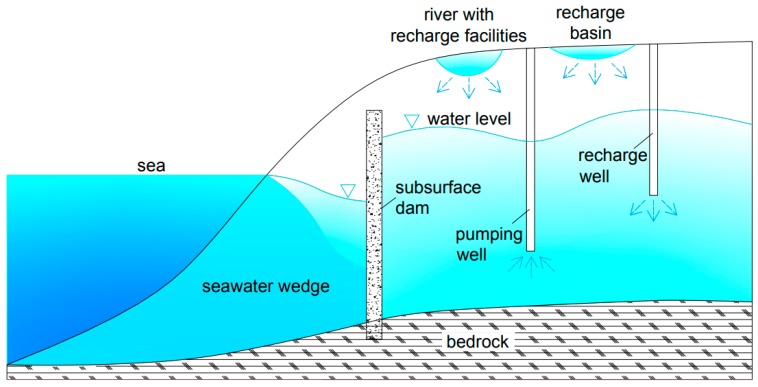
A sketch map of the subsurface dam.

The layout of the artificial underground reservoir is presented in Figure 3. The project mainly consists of the subsurface dam, artificial recharge system, and groundwater extraction facilities. (1) Subsurface dam. The 13.95 km-long subsurface dam is made of concrete, with a thickness of >0.18 cm and a permeability coefficient of <1 × 10^−6^ cm/s. The dam rests on the impervious stratum, and the crest is at such a depth to allow groundwater overflow. The sketch map of the subsurface dam is shown in Figure 4. (2) Artificial recharge system. Surface recharge methods include a recharge basin and cascade water retaining dams in the Wanghe River. Subsurface recharge techniques consist of 1068 recharge wells and 187 trenches in the Wanghe River and 142 recharge wells in the delivery canal connecting the Wanghe River and the recharge basin. (3) Groundwater extraction facilities. The stored water is extracted from two well batteries along the Wanghe River, which provide water for the water treatment plant and irrigation wells scattered in the reservoir area. 

### 2.3. Groundwater Sampling and Analysis

Isotopic tracers have been used in characterizing groundwater movement, age, and recharge rate. 31 groundwater samples for tritium were collected in April 2017, 17 of which within the reservoir and 14 in the downstream area (Figure 3). The detailed information on tritium sampling wells including well depth, aquifer characteristics, and surrounding environment were recorded on the spot. Water sampling was conducted according to ‘Groundwater Quality testing methods (DZ/T 0064-1993), Ministry of geology and mineral resources of the People’s Republic of China’. Sampling site location and groundwater depth were recorded on the spot. Tritium concentration was measured by 1220 Quantulus Ultra Low Level Liquid Scintillation Spectrometer (Perkin Elmer, Waltham, MA, USA) according to ‘Analytical method of tritium in Water (GB12375-1990), State Environmental Protection Administration of China’.

Filed investigations of groundwater nitrogen contamination in the research area were conducted in July 2014, April 2015, and August 2015. The location of the sampling sites can be seen in Figure 3. The sampling wells included groundwater level monitoring wells and irrigation wells, and all the wells are constructed in the unconfined aquifer. Water sample collection and preservation were conducted under the guidance of ‘Water Quality-Technical regulation of the preservation and handling of samples (HJ493-2009), the Ministry of Environmental Protection of the People’s Republic of China’. Multi 340i portable water quality analyzer (WTW, Munich, Germany) was employed to detect the temperature, salinity, dissolved oxygen (DO), conductivity (EC), and pH of water samples on site. The determination of ${\mathrm{NO}}_{3}^{-}$-N, ${\mathrm{NO}}_{2}^{-}$-N, and ${\mathrm{NH}}_{4}^{+}$-N was performed in accordance with ‘Technical Specifications for Environment Monitoring of Groundwater (HJ/T164-2004), The Ministry of Environmental Protection of the People’s Republic of China (MEP)’. ${\mathrm{NO}}_{3}^{-}$-N was determined by the cadmium reduction method, ${\mathrm{NO}}_{2}^{-}$-N was measured by N-(1-naphthyl)-ethylenediamine spectrophotometry, and ${\mathrm{NH}}_{4}^{+}$-N was determined by the salicylic acid–hypobromite oxidation method.

### 2.4. Sources of Nitrogen Pollution in Stored Water

#### 2.4.1. Agricultural Pollution

The main cropping system in the reservoir area is winter wheat–summer maize rotation, which is a high-input and high-yield agricultural production system characterized by choosing good seed varieties, precision seeding, full irrigation, heavy fertilization, and a high level of mechanization. The average amount of nitrogen fertilizer applied reaches 850 kgN/ha/a. Winter wheat requires 240 kg/ha chemical fertilizer-N and 250 kg/ha organic fertilizer-N in the whole growth stage. For summer wheat, the amount of chemical fertilizer-N is 240 kg/ha. In addition, nitrogen fertilizer is applied after harvesting to promote straw decomposition. Table 1 summarizes the schedule of the nitrogen fertilizer applications during the winter wheat and summer maize growth period.

The precipitation is principally concentrated over June to September in a few heavy rain events, which synchronizes with the growth period of summer maize. In addition, winter maize requires 2000 m^3^/ha irrigation water throughout the growing period. Conventional irrigation methods, including flood and furrow irrigation, have been predominantly practiced. The intensive and heavy rainfall and unreasonable irrigation lead to loss of nitrogen in surface runoff and soil nitrogen movement out of the effective crop root zone to the deep soil profile and groundwater. In north China, N leaching loss is estimated to be 25% of the total applied N in the summer maize-winter wheat rotation system [41]. The contribution of agriculture as a source of groundwater N pollution is about 873.25 t/a.

#### 2.4.2. Domestic Pollution

There are many village clusters in the artificial underground reservoir area, and the total population is about 41,500. Most of the villages are uncovered sewage collection pipe networks and treatment plants, and household-based decentralized wastewater treatment is the most commonly adopted method. Wastewater from kitchens and bathrooms and excreta from toilets are collected and preliminarily treated in septic tanks. The sludge is returned to the crop field as high-quality organic fertilizers, and the septic tank effluent is usually discharged directly to the nearby ponds through open trenches without any subsequent processes. According to a survey of 50 rural households in the reservoir area, the volume of effluent averages 60 L/pers/d, and the concentrations of TN and ${\mathrm{NH}}_{4}^{+}$-N are 80 mg/L and 35 mg/L, respectively. The amount of N derived from septic tanks is 72.71 t/a, by a rough estimate.

#### 2.4.3. Livestock and Poultry Farming Pollution

There are several small-sized livestock and poultry farms scattered in the reservoir area, with 500 pigs, 200 beef cattle, and 20,000 chickens. The farms are generally equipped with a dry manure collection system, in which the feces and urine are diverted at the point of excretion. The dry excrement is collected to produce organic fertilizer, while urine and flushing water are generally discharged directly into groundwater. Table 2 lists the amount of livestock wastewater of different farmed species and concentrations of TN and ${\mathrm{NH}}_{4}^{+}$-N. The load of N from livestock and poultry farming activity is estimated to be 1.82 t/a.

## 3. Results and Discussion

### 3.1. Groundwater Flow Field Change and Water Level Fluctuation

**Figure 5:**
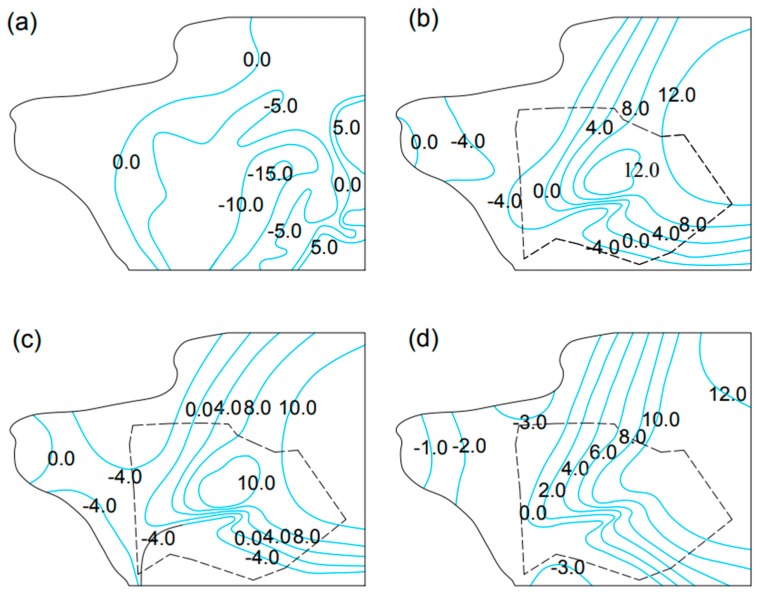
Groundwater flow field in (**a**) 1994/06/01 before reservoir construction; (**b**) 2004/06/01 after reservoir construction; (**c**) 2009/06/01 after reservoir construction; (**d**) 2016/06/01 after reservoir construction. (unit: m).

Figure 5 presents the groundwater flow change in the study area before and after artificial underground reservoir construction. On 1 June, 1994, there was a cone of depression in the reservoir area. After the reservoir was constructed, the cone disappeared, and the reservoir water level recovered rapidly. At the same time, a groundwater level decline in the downstream area after reservoir construction can be observed.

**Figure 6:**
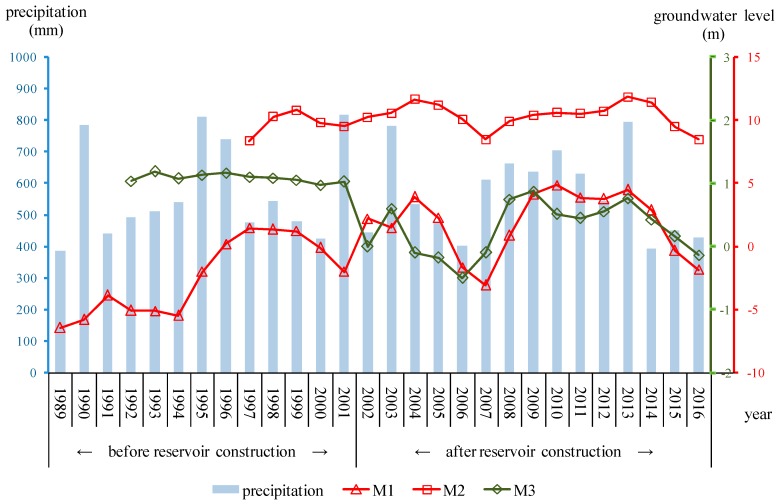
Relationship between annual precipitation and average annual groundwater level.

The long-term groundwater level monitoring data of three observation wells in the research area were used to analyze groundwater level fluctuation before and after reservoir operation (Figure 3). M1 and M2 were located in the reservoir, and M3 was in the downstream region. Figure 6 describes the annual precipitation and annual mean groundwater level of the monitoring wells. The observation period of well M1 was from 1989 to 2016. The groundwater level in M1 was strongly influenced by the construction of the artificial underground reservoir. After the construction of the reservoir in 2001, the groundwater level rose significantly from –2.00 m to 2.20 m in 2002, despite the drop of rainfall from 818.4 mm 444.8 mm. the groundwater level of well M1 still showed a rising trend, and the average groundwater level was −2.43 m from 1989 to 2001 and 1.85 m from 2002 to 2016. The average annual groundwater level changed consistently with annual precipitation in well M2, which had a shallow water depth. By reviewing the variation of groundwater level of M2, it can be easily found that the groundwater level did not evidently change with the construction of the reservoir. The likely scenario was that the observation well was located far from the subsurface dam, and, hence, it was less affected. The observation period of well M3 was 1992–2016. The trend of groundwater level correlated to the rainfall. Before the construction of the reservoir, the groundwater level was relatively stable and averaged 1.08 m from 1992 to 2001. After the operation of the project, the water level decreased dramatically from 1.03 m to 0.0 m in 2002 and averaged 0.27 m from 2002 to 2016. In the offshore area, groundwater has been extracted by scattered pumping wells for irrigation and land-based marine aquaculture. The water level decline in M3 can be explained by reduced groundwater recharge after reservoir construction. According to field investigation and data from the local statistical bureau, groundwater consumption for land-based marine aquaculture has remained stable since 1990. Due to cultivated land degradation, agricultural water consumption decreased gradually. This suggests that the subsurface dam deprived the downstream aquifer of the runoff that it received under the natural flow regime.

### 3.2. Groundwater Tritium Level and Renewal Rate

**Figure 7:**
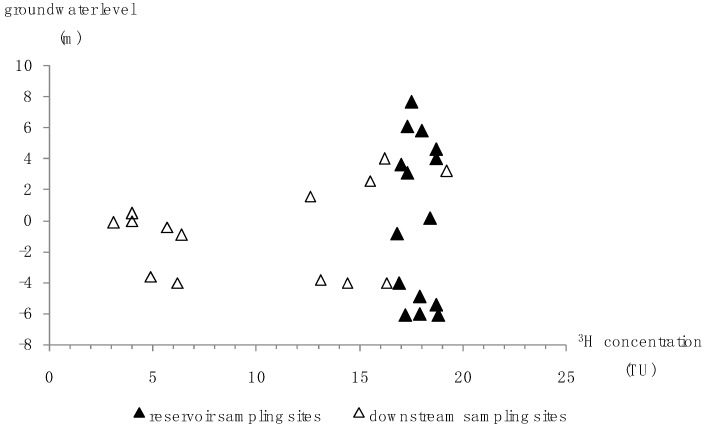
Relationship between groundwater level and tritium concentration.

**Figure 8:**
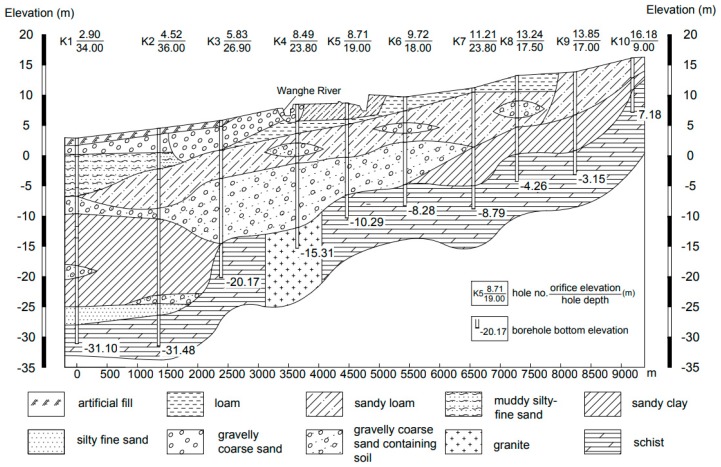
Hydrogeological section of the storage layers (line A-A’ in Figure 3).

**Figure 9:**
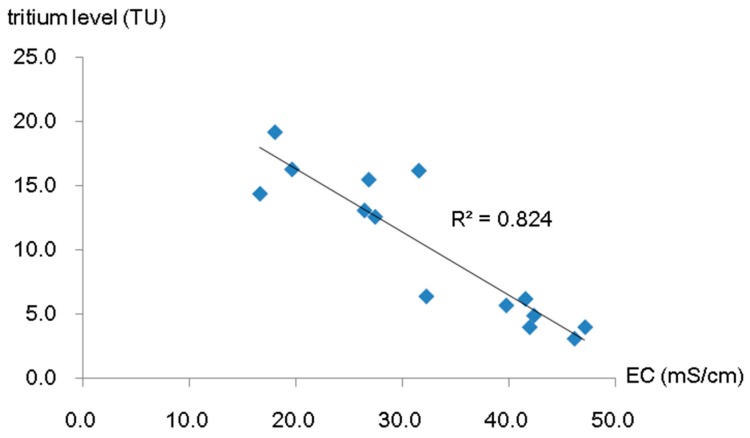
Relationship between groundwater conductivity (EC) and tritium concentration.

Groundwater tritium levels are displayed in Table 3, and the relationship between the concentration of tritium and groundwater level is plotted in Figure 7. The reservoir tritium concentration changed within a narrow range of 16.8–18.8 TU, and there was no decreasing trend of tritium concentration with groundwater depth. Given that the groundwater sampling wells varied in depth (Table 3), this confirmed that the stored water was well-mixed in both horizontal and vertical directions. According to our investigation, the construction of the subsurface dam, the accelerated groundwater flow caused by pumping, and the good hydraulic connection between storage layers contributed to groundwater mixing and the homogeneous tritium distribution. With the construction of the dam, the artificial underground reservoir formed a relatively closed space for water storage. The amount of groundwater withdrawal for irrigation of crops and plants is approximate to 2.355 × 10^6^ m^3^ per year, which occupies 41.4% of the total reservoir capacity. The massive pumping accelerated groundwater flow and promoted water mixing. Furthermore, as depicted in Figure 8, the storage aquifer system of the artificial underground reservoir has 3–4 layers and a total effective thickness of 5–16 m. The storage layers are mainly composed of quaternary alluvial coarse-grained sand and gravel interbedded with thin fine marine deposits, and there is a high degree of hydraulic connection between the storage layers. Under the above conditions, the uniform tritium distribution in the reservoir is highly reasonable. In the downstream region, tritium concentration varied significantly between 3.1 and 19.2 TU. A clear downtrend of tritium concentration towards the sea was observed. Tritium closer to shore was at a low level, which was presumed to be the result of seawater intrusion. The correlation analysis between groundwater EC and tritium concentration in the downstream region (sampling sites T18–T31) suggested that it was caused by seawater intrusion at a significant level (Figure 9). 

As the artificial underground reservoir is mainly recharged through flash floods originating from rainfall events, the use of ^3^H to determine the groundwater renewal rate requires detailed knowledge of tritium concentration in atmospheric precipitation. Since there is no coverage of GNIP (the Global Network Isotopes in Precipitation) stations for ^3^H content in precipitation in the study area, a multi-layer neural network employing a Backpropagation Artificial Neural Network (BP-ANN) was used to reconstruct the annual mean tritium concentration in precipitation [42,43]. The neural network toolbox in MATLAB was used for network training. Nine-hundred and fourty-six sets of data from 62 GNIP stations [44] located between 25° N and 75° N were selected and are randomly divided into 850 Training datasets and 96 Test datasets.

**Figure 10:**
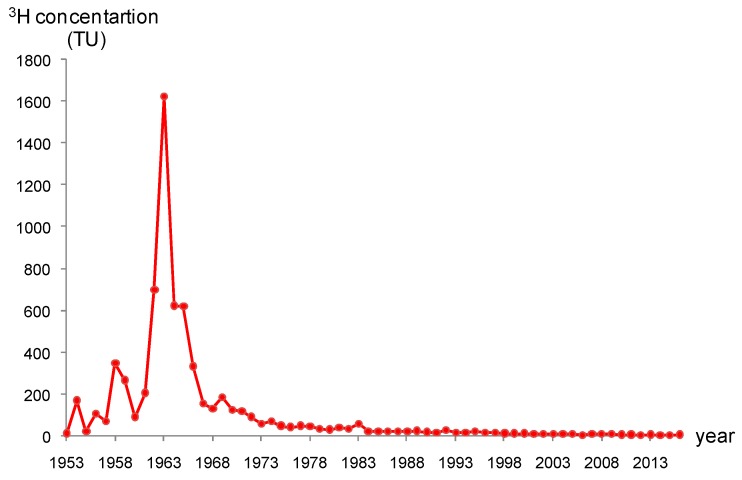
Reconstructed tritium concentration series in precipitation in the study area.

BP-ANN is composed of an input layer, a hidden layer and an output layer. The input layer contains six neural cells (longitude, latitude, altitude, yearly precipitation, mean annual temperature, and particular year), which are impact factors associated with tritium content in precipitation. The output layer is the annual mean tritium concentration in precipitation. The hidden layers were automatically generated by MATLAB. The BP-ANN network was created after training. The maximum and mean relative errors between the reconstructed and measured values of the 96 test data set were 10.17% and 7.24%, respectively, indicating the effectiveness of the BP-ANN method in generating tritium content in precipitation. The ^3^H concentration series in the study area reconstructed with the trained BP-ANN model is presented in Figure 10.

The groundwater renewal rate was calculated based on the well-mixed reservoir model [45,46]. The model assumes that a complete mixing of groundwater issued from successive recharge events occurs within the aquifer. The groundwater tritium content (at annual time-step) can be calculated from its radioactive decay and annual input as follows:

^3^H*_gwi_* = (1 − R*_i_*)^3^H*_gwi-1_*e^−λ^ + R*_i_*^3^H*_0i_* (1)

where ^3^H*_gwi_* is the tritium content in groundwater in year *i*; ^3^H_gwi-1_ is the tritium content in groundwater in year *i-1*; *^3^H_0i_* is the tritium content in input water in year *i*; *R_i_* is the annual renewal rate in year *i*; *λ* is the radioactive decay constant, with *λ* = 0.05626/*a*.

The calculation starts from year 1952, when an atmosphere nuclear explosion was conducted. Before 1952, groundwater tritium content was constant and set to be 10 TU. Then, tritium content in 1952 can be obtained by

^3^H*_gw1952_* = ^3^H_0_/(*λ*/R + 1) (2)

where *R* is the mean groundwater renewal rate.

By assuming that annual recharge is in related to annual precipitation, *R_i_* is further modified to be

R*_i_* = R (P*_i_* − P*_t_*)/(P*_m_* − P_t_) (3)

where *P_i_* is the annual precipitation in year *i, P_m_* is the annual mean precipitation, and P*_t_* is the minimum limit precipitation, *mm/a*.

**Figure 11:**
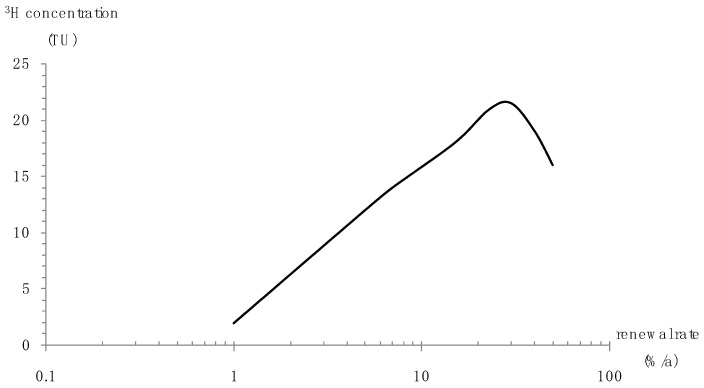
Relationship between groundwater tritium concentration and groundwater renewal rate.

Substituting the time series of precipitation and recovered precipitation tritium concentration into Equation (1), the relationship between groundwater tritium concentration and groundwater renewal rate is obtained (Figure 11).

The groundwater renewal rates of the groundwater tritium sampling points are listed in Table 3. The average groundwater renewal rate of the stored water ranged from 14% to 16%/a and averaged 14.8%/a. The groundwater renewal rate in the downstream was from 2% to 16%/a. 

### 3.3. Groundwater Nitrogen in the Reservoir Area

It is regrettable that there is a lack of long-term continuous observation of groundwater nitrogen in the research area. However, the distribution and change of nitrogen in groundwater exhibited some regularity as is evidenced by the data collected.

Table 4 illustrates the ratios of ${\mathrm{NO}}_{3}^{-}$-N, ${\mathrm{NO}}_{2}^{-}$-N, and ${\mathrm{NH}}_{4}^{+}$-N to dissolved inorganic nitrogen (DIN) in stored and downstream groundwater. From this table, it can be easily observed that ${\mathrm{NO}}_{3}^{-}$-N was the predominant form of dissolved inorganic nitrogen (DIN) in the reservoir area, accounting for, respectively, 99.57%, 98.18%, and 99.75% of DIN in July 2014, April 2015, and August 2015. Based on the previous calculations in 2.4, agriculturally derived nitrate is the major source of groundwater nitrate in the stored water. After nitrogen fertilizer is applied, ammonium ions are strongly adsorbed by the topsoil, which leads to the reduction of ${\mathrm{NH}}_{4}^{+}$-N concentration with soil depth. However, unlike ammonium ions, nitrate shows negligible adsorption to the soil, and is, therefore, highly mobile. Nitrogen in the form of ${\mathrm{NO}}_{3}^{-}$-N is susceptible to leaching beyond the root zone into deep soil layers and groundwater. Water samples were collected half a month after the last nitrogen fertilizer application in July 2014 and August 2015, and the interval between water sampling in April 2015 and the most recent nitrogen fertilization was about 6 months. The high concentration of ${\mathrm{NO}}_{3}^{-}$-N and relatively low or undetected concentrations of ${\mathrm{NO}}_{2}^{-}$-N and ${\mathrm{NH}}_{4}^{+}$-N indicated that the pollution occurred a considerable time ago, and self-purification has been substantially completed.

**Figure 12:**
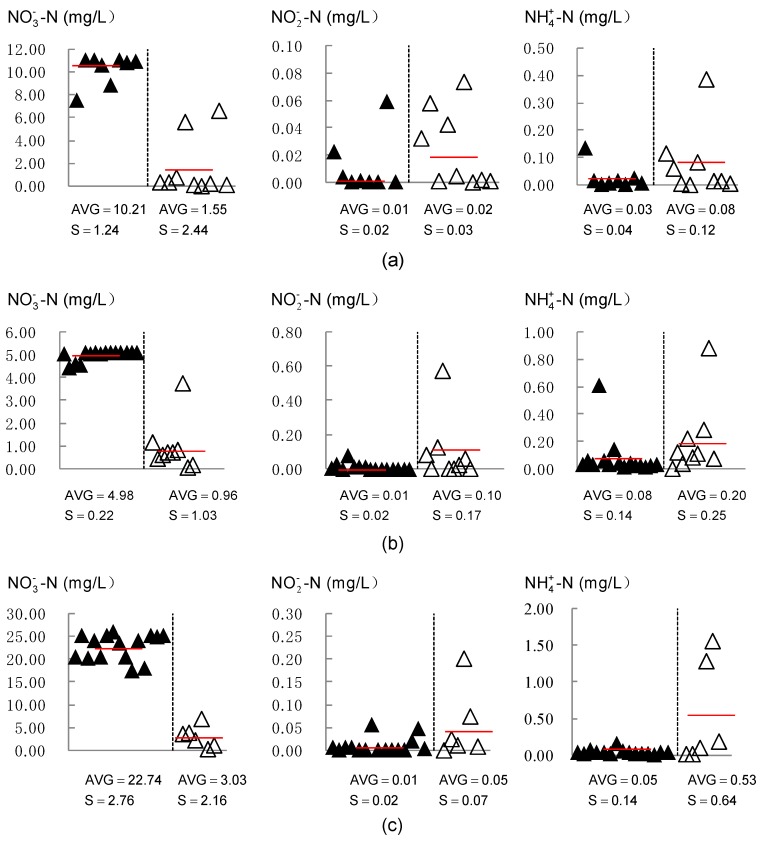
Groundwater ${\mathrm{NO}}_{3}^{-}$-N, ${\mathrm{NO}}_{2}^{-}$-N, and ${\mathrm{NH}}_{4}^{+}$-N concentrations in stored water and the downstream area in (**a**) July, 2014, (**b**) April, 2015, and (**c**) August, 2015 (▲ reservoir sampling site; △ downstream sampling site).

Figure 12 depicts groundwater ${\mathrm{NO}}_{3}^{-}$-N, ${\mathrm{NO}}_{2}^{-}$-N, and ${\mathrm{NH}}_{4}^{+}$-N concentrations in the reservoir and the downstream area. It appeared such an interesting phenomenon that groundwater nitrate was distributed evenly in the reservoir area, which coincided with the distribution of tritium. According to the analysis above, the homogeneous distribution of nitrate was attributed to the combined action of the subsurface dam, land use pattern, and artificial groundwater flow caused by extensive pumping of groundwater. Agricultural land is the prevailing land use in the reservoir area, and nitrogen fertilizer leaching is the largest source of groundwater nitrate contamination. Good hydraulic connectivity and rapid groundwater movement caused by pumping facilitated nitrate transportation and equalized its distribution in the reservoir. A similar observation was documented in the study of the nitrate contamination of the Sunagawa artificial underground reservoir by Ishida, S. et al. [36,37]. Due to pumping a large amount of groundwater for irrigation, an extensive uniform area of ${\mathrm{NO}}_{3}^{-}$-N concentration in the Sunagawa artificial underground reservoir was developed.

**Figure 13:**
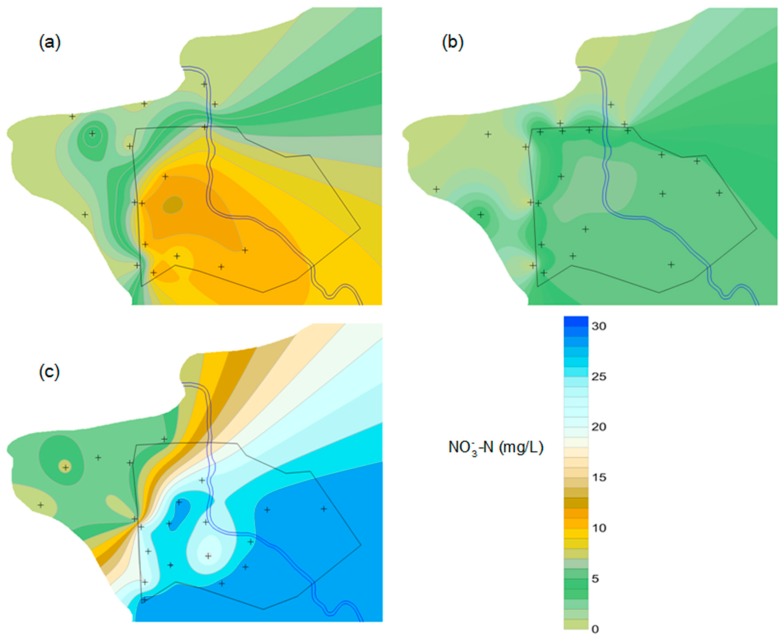
The variation of the distribution of groundwater nitrate concentration with time. (**a**) groundwater ${\mathrm{NO}}_{3}^{-}$-N level in July, 2014; (**b**) groundwater ${\mathrm{NO}}_{3}^{-}$-N level in April, 2015; (**c**) groundwater ${\mathrm{NO}}_{3}^{-}$-N level in August, 2015.

Groundwater nitrate concentration in the reservoir varied considerably over time, as highlighted in Figure 13. ${\mathrm{NO}}_{3}^{-}$-N concentration in stored water ranged from 7.49 to 11.00 mg/L, and the average level was 10.56 mg/L in July 2014. In April 2015, the value decreased to 4.58–5.54 mg/L, with a mean value of 5.36 mg/L. High nitrate content was observed in the study area in August, 2015, which ranged from 17.47 to 25.41 mg/L and averaged 22.74 mg/L. This level far exceeded the threshold value for drinking purposes recommended by the World Health Organization (10 mg/L). Groundwater sampling in April, 2015 was conducted before the jointing-stage topdressing of winter wheat, over half a year after basal dressing in October of the previous year. Due to the poor rainfall during this period, there was no possibility that the low ${\mathrm{NO}}_{3}^{-}$-N concentration was caused by dilution, and denitrification was responsible for the consumption of nitrate. Due to the synchronization of the maize growth period and rainy season, the massive nitrogen loss through leaching led to nitrate retention and accumulation in groundwater. Therefore, the groundwater nitrate concentration was the highest in August 2015, followed by that in July 2014. Dong, W.L. et al. [47] also found that in areas where an agricultural non-point source is the leading contributor to groundwater nitrate pollution, ${\mathrm{NO}}_{3}^{-}$-N concentration in the shallow groundwater at the end of the rainy season is greater than that at the end of the dry season. This suggests that seasonal variation of nitrate content should be taken into consideration when conducting groundwater nitrate pollution investigation and evaluation. 

Riverine groundwater ${\mathrm{NO}}_{2}^{-}$-N and ${\mathrm{NH}}_{4}^{+}$-N concentrations in the flush time (July, 2014) of the Wanghe River were comparatively higher. However, the high-concentration zone was non-existent in April 2015 and August 2015 when the river ran dry. This indicated that river pollution could probably be one of the major sources of groundwater ${\mathrm{NO}}_{2}^{-}$-N and ${\mathrm{NH}}_{4}^{+}$-N. River seepage serves as one of the main sources of groundwater recharge and groundwater replenishment is greatly enhanced by artificial recharge facilities in the river course. The groundwater ${\mathrm{NO}}_{2}^{-}$-N and ${\mathrm{NH}}_{4}^{+}$-N level rose in the immediate vicinity of the river through mixing with polluted river water as the aquifer captured water from the river. Another condition that justified the high-concentration zone was the livestock and poultry farms located near the river, where sewage was generally discharged on site and seeped into the ground.

### 3.4. Groundwater Nitrogen in Downstream Area

The groundwater nitrogen in the downstream area was very different from that inside the reservoir. As can be seen in Figure 13, an abrupt decrease in groundwater ${\mathrm{NO}}_{3}^{-}$-N concentration was observed at the dam site. This provided strong evidence to suggest nitrate enrichment in stored water. By observing the distribution of nitrogen in the downstream area, it can be easily found that the groundwater nitrate in the farming areas near the subsurface dam was higher and showed a declining trend from inland to the coast. However, the time variation of downstream groundwater ${\mathrm{NO}}_{3}^{-}$-N concentration was consistent with that in the reservoir area. The highest level of ${\mathrm{NO}}_{3}^{-}$-N concentration in the downstream area occurred also in August 2015, which varied from 0.32 to 6.95 mg/L and averaged 3.03 mg/L. In July 2014, the range was from 0.02 to 6.55 mg/L and the mean value was 1.55 mg/L. The downstream ${\mathrm{NO}}_{3}^{-}$-N concentration was at its lowest in April 2015, with a range of 0.07–3.75 mg/L and an average of 0.96 mg/L. The ratio of ${\mathrm{NO}}_{3}^{-}$-N to DIN decreased towards the sea, and the dominant component was ${\mathrm{NH}}_{4}^{+}$-N in the aquiculture areas.

Intensive farming forms the bulk of aquiculture production along the coast of Bohai Bay, which involves high level inputs of feed and fertilizer, as well as high stocking density. The effluent from aquafarms is not recirculated and treated within the farming operation but is discharged directly to the sea through pipelines or open trenches. Aquaculture wastewater contains high-strength ${\mathrm{NH}}_{4}^{+}$-N, which comes from surplus bait and fishery fertilizer, metabolites and excreta from farmed animals, and the decomposition of biological debris. In addition, due to the extensive use of disinfectants, and the constant threat of oxygen deficiency, the nitrification is severely hindered; hence, ammonia and nitrite accumulation takes place. As some of the earthen ponds and drainage ditches were designed without anti-seepage treatment, the wastewater enriched in ${\mathrm{NO}}_{2}^{-}$-N and ${\mathrm{NH}}_{4}^{+}$-N easily penetrated the groundwater. Aquaculture pollutants may migrate upstream with saltwater. Kang, P.P. et al. [48] identified the sources of groundwater nitrate in the inland area downstream the Wanghe artificial underground reservoir using a dual-stable isotope method and found that nitrogen derived from mariculture and fertilizer accounted for the major part of the groundwater nitrate. The contribution rate of nitrate derived from mariculture decreased towards the inland while that from fertilizer increased. Mariculture derived nitrate was detected in downstream sampling wells close to the subsurface dam. The subsurface dam could protect the stored water from being contaminated by downstream pollutants.

## 4. Conclusions

This study conducted field surveys in July 2014, in April, 2015, August, 2015 and April, 2017 to investigate the impact of the Wanghe artificial underground reservoir on groundwater flow and quality in the local and downstream areas. This analysis leads to a conclusion that the artificial underground reservoir interfered in groundwater circulation and resulted in nitrate accumulation in the stored water.

Long-term groundwater level monitoring data revealed that the groundwater level near the dam site rose after reservoir construction. The downstream groundwater level showed a decreasing trend, suggesting that the reservoir deprived the downstream aquifer of the runoff, which it received under the natural flow regime. Isotopic evaluation using ^3^H based on the well-mixed reservoir model indicated that the groundwater tritium in the stored water was well-mixed in both horizontal and vertical directions, which was caused by the construction of the subsurface dam, artificially-enhanced groundwater flow due to extensive pumping, and a good hydraulic connection between storage layers. The human intervention on water circulation also made a difference in the groundwater renewal rate between stored and downstream water.

The groundwater ${\mathrm{NO}}_{3}^{-}$-N concentration was significantly higher in the reservoir compared to that in the downstream, and the agriculture non-point source was the single largest nitrate source in the reservoir water. Groundwater nitrate was distributed homogeneously in the reservoir area, which was attributed to the construction of the subsurface dam, land use patterns, and artificial groundwater flow caused by extensive pumping. The nitrate level varied considerably in all three field surveys due to rainfall variety and fertilization patterns.

It is necessary to find ways to seek balance and compromises between upstream and downstream interests and ecosystems, though a high priority is given to the upstream water users. The semi-pervious subsurface barriers implemented on Miyako-jima Island, Japan, and in Shahrekord, Iran, serve as good practice, allowing part of the seepage of stored water to the downstream aquifer to avoid concentration of agricultural chemicals. Water quality is key to the sustainable operation of an artificial underground reservoir, which was threatened by over-fertilization and inadequate disposal of domestic and livestock waste. Improving irrigation and fertilization strategies can be a solution to groundwater nitrate accumulation. The downstream area was subjected to groundwater flow reduction, which aggravated the seawater intrusion and upstream movement of aquaculture pollutants. Therefore, groundwater exploitation should be controlled, and recirculating aquaculture systems should be promoted to protect limited fresh and brackish water resources.

It should be acknowledged that there is a lack of long-term groundwater quality monitors in the study area to detect the trend of water quality changes. Another limitation of this study is that at least monthly data should be collected to evaluate water pollution, as ${\mathrm{NO}}_{3}^{-}$ concentration fluctuates greatly within the seasons.

## Figures and Tables

**Table 1:** Nitrogen fertilizer application calendar.

Calendar	Growth Period		Agricultural Practices	N-Fertilizer Application (kgN/ha)
June 10th	summer maize	sowing period	summer maize sowing	/
July 10th		jointing period	top application	80 (chemical fertilizer N)
August 1st		booting period	top application	120 (chemical fertilizer N)
August 20th		filling period	top application	40 (chemical fertilizer N)
October 1st		harvest period	summer maize harvest straw mulching and nitrogen fertilizer application	60 (chemical fertilizer N)
October 5th	winter wheat	sowing period	winter wheat sowing base fertilizer application	250 (organic fertilizer N) 120 (chemical fertilizer N)
April 20th		jointing period	top application	120 (chemical fertilizer N)
June 5th		harvest period	winter wheat harvest straw mulching and N-fertilizer application	60 (chemical fertilizer N)

**Table 2:** Amount of livestock wastewater and TN, ${\mathrm{NH}}_{4}^{+}$-N concentrations.

Livestock Species	Amount of Wastewater (L/d/pd)	N Concentration (mg/L)	
		TN	${\mathrm{NH}}_{4}^{+}$-N
swine	10	400	250
beef cattle	30	80	50
chicken	0.25	500	200

**Table 3:** Groundwater tritium concentration and renewal rate.

Sampling Site	In/Downstream the Reservoir	Ground Elevation (m)	Groundwater Level (m)	Well Depth (m)	Aquifer Characteristics	Surrounding Environment	EC at 25 ℃ (mS/cm)	^3^H Level (TU)	Groundwater Renewal Rate (%/a)
T1	in	3.4	3.1	8	unconfined	wasteland	1.82	17.3	14
T2	in	4.2	3.6	3	unconfined	cultivated land, near the river	4.91	17.0	14
T3	in	4.6	4.0	22	unconfined	cultivated land	4.16	18.7	16
T4	in	5.0	–4.9	about 25	unconfined	residential land	1.79	17.9	15
T5	in	6.5	4.6	31	unconfined	vegetable field	1.78	18.7	16
T6	in	9.0	6.1	35	unconfined	residential land, near the river	1.04	17.3	14
T7	in	5.5	0.2	about 35	unconfined	cultivated land	3.84	18.4	15
T8	in	10	5.8	38	unconfined	cultivated land	4.80	18.0	15
T9	in	6.0	–4.0	about 40	unconfined	residential land	2.52	16.9	14
T10	in	5.2	–5.4	25	unconfined	cultivated land	1.50	18.7	16
T11	in	10.0	–0.8	20	unconfined	cultivated land, near the river	1.66	16.8	14
T12	in	13.1	–6.1	35	unconfined	cultivated land	3.55	17.2	14
T13	in	16.0	7.7	18	unconfined	orchard	1.73	17.5	15
T14	in	4.0	–6.0	about 20	unconfined	cultivated land	4.61	17.9	15
T15	in	13	3.6	about 35	unconfined	cultivated land	2.28	17.0	14
T16	in	9.0	–6.1	35	unconfined	orchard	1.88	18.8	16
T17	in	7.5	–9.1	14	unconfined	cultivated land	1.77	16.8	14
T18	downstream	0.7	0.5	26	unconfined	aquaculture land, near the river	41.9	4	2
T19	downstream	4.4	1.6	11	unconfined	cultivated land	27.4	12.6	6
T20	downstream	3.2	2.6	10	unconfined	waste land	26.8	15.5	9
T21	downstream	4.2	4.0	about 20	unconfined	residential land	31.5	16.2	11
T22	downstream	4.5	3.2	20	unconfined	cultivated land	18.0	19.2	16
T23	downstream	0.2	0.0	12	unconfined	aquaculture land	47.1	4	2
T24	downstream	0.2	–0.1	30	unconfined	aquaculture land	46.1	3.1	2
T25	downstream	5	–4.0	23	unconfined	livestock farm	19.6	16.3	12
T26	downstream	3.2	–0.9	22	unconfined	pine forest	32.2	6.4	3
T27	downstream	5	–3.8	18	unconfined	wasteland	26.4	13.1	6
T28	downstream	3.7	–0.4	27	unconfined	aquaculture land	39.7	5.7	3
T29	downstream	4.0	–4.0	18	unconfined	residential land	16.6	14.4	8
T30	downstream	7.4	–4.0	about 40	unconfined	aquaculture land	41.5	6.2	3
T31	downstream	6	–3.6	33	unconfined	aquaculture land	42.3	4.9	2

**Table 4:** Ratios of ${\mathrm{NO}}_{3}^{-}$-N, ${\mathrm{NO}}_{2}^{-}$-N and ${\mathrm{NH}}_{4}^{+}$-N to dissolved inorganic nitrogen (DIN) in stored and downstream groundwater (%).

Form of Inorganic-N	2014.07				2015.04				2015.08			
	Inside		Downstream		Inside		Downstream		Inside		Downstream	
	Range	Average	Range	Average	Range	Average	Range	Average	Range	Average	Range	Average
${\mathrm{NO}}_{3}^{-}$-N	97.92–99.97	99.57	3.91–99.73	76.32	86.83–99.65	98.18	0.33–99.20	64.52	99.17–99.91	99.75	16.42–99.24	79.46
${\mathrm{NO}}_{2}^{-}$-N	0.00–0.54	0.12	0.04–15.56	4.69	0.04–1.52	0.23	0.26–36.93	6.89	0.00–0.22	0.04	0.03–2.83	1.33
${\mathrm{NH}}_{4}^{+}$-N	0.02–1.79	0.31	0.00–80.54	18.99	0.29–11.65	1.59	0.27–99.41	28.59	0.07–0.62	0.21	0.42–79.45	19.21

## Appendix A

**Table A1:** Basic information of some artificial underground reservoirs in China.

Reservoir Name	Reservoir Area (km^2^)	Total Storage Capacity (10^3^m^3^)	Active Storage (10^3^m^3^)	Reservoir Structure	Construction Method of Cut-Off Wall	Vertical Configuration of Cut-Off Wall	Wall Length (m)	Seepage Control Area of Cut-Off Wall (10^3^ m^2^)	Construction Period	Location
Nangong	206	460,000	84,000	plain palaeochannel	NAp	NAp	NAp	NAp	1977–1982	Hebei
Wanghe	68.49	56,930	32,730	coastal alluvial plain	high pressure jet grouting	keyed-into low permeability layer	13,593	27.2	1999–2006	Shandong
Jiahe	63.26	205,200	65,000	intermontane valley alluvial plain	high pressure jet grouting	keyed-into low permeability layer	2511	4.5	2000–2001	Shandong
Daguhe	421.69	384,130	237,800	intermontane valley alluvial plain	high pressure jet grouting, geomembrane wall, permeation grouting	keyed-into low permeability layer	4350	3.5	1997–1998	Shandong
Hutuohe	436.5	100.4	NAp	alluvial fan, cone of depression	NAp	NAp	NAp	NAp	2012–2014	Hebei
Longhe *	8.5	865	NA	intermontane valley alluvial plain	high pressure jet grouting	keyed-into low permeability layer	544.6	0.5	1998–2000	Liaoning
Jianbaohe	26.09	6904.1	6525.2	intermontane valley alluvial plain	high pressure jet grouting	keyed-into low permeability layer	930	1.5	2001–2003	Liaoning
Huangshuihe	53	53,590	39,290	coastal alluvial plain	high pressure jet grouting	keyed-into low permeability layer	5996	16	1993–1995	Shandong
Balishahe	0.7	398	355	coastal alluvial plain	high pressure jet grouting	keyed-into low permeability layer	756	5500	1988–1989	Shandong
Maguan	NAp	1325.42	1325.42	karst subterranean river (surface–subsurface reservoir)	damming underground river	NAp	NAp	NAp	1990–1990	Guizhou
Pengbao	75	84,380	7280	cone of depression	plastic concrete slurry wall, permeation grouting grouting	keyed-into low permeability layer	4050	16.2	2009–2011	Ningxia
Liangchenghe	12.3	23,050	10,600	coastal alluvial plain	deep soil mixing	keyed-into low permeability layer	3990	4.8	2016–2017	Shandong
Rushanhe	19.6	21,970	16,860	coastal alluvial plain	high pressure jet grouting	keyed-into low permeability layer	7600	11.4	2017–2018	Shandong
Shilin	NAp	6725.5	5508.7	karst subterranean river (surface-subsurface reservoir)	permeation grouting	keyed-into low permeability layer	4840	10	2012–2013	Yunnan
Shangba	NAp	2800	2680	karst subterranean river	damming underground river	keyed-into low permeability layer	NAp	NAp	2006–2007	Guizhou
Wulichong	NAp	79,490	79,490	karst subterranean river (surface-subsurface reservoir)	high pressure jet grouting, damming underground river, reinforced concrete slurry wall, damming at outfall of underground river	hanging wall	1383	26.2	1991–1996	Yunnan
Baixi	NA	NA	NA	coastal alluvial plain	high pressure jet grouting	keyed-into low permeability layer	514	8153	2009–2009	Zhejiang
Sanguanmiao	1	638.1	1472.46	intermontane valley alluvial plain	high pressure jet grouting	keyed-into low permeability layer	NA	NA	2001–2002	Liaoning
Laolongwan	NA	NA	360	intermontane valley alluvial plain	geomembrane	keyed-into low permeability layer	391.4	NA	1995–1998	Liaoning

*: Longhe artificial underground reservoir was abandoned due to water contamination. NAp: information not applicable; NA: information not available
